# A Case Report of an Atypical Presentation of Morvan Syndrome

**DOI:** 10.7759/cureus.68879

**Published:** 2024-09-07

**Authors:** Sara G Haroutunian, Rana Hoseen, Donya Farmand, Marinelle Camilon, Sanaz Hashemi

**Affiliations:** 1 Medicine, Ross University School of Medicine, Bridgetown, BRB; 2 Internal Medicine, California Hospital Medical Center, Los Angeles, USA; 3 Medical Genetics, National Institutes of Health, Bethesda, USA; 4 Family Medicine, California Hospital Medical Center, Los Angeles, USA

**Keywords:** amphiphysin, blepharospasm, morvan’s fibrillary chorea, morvan syndrome, muscle spasm, myokymia, paraneoplastic neurological syndromes, voltage-gated potassium channel

## Abstract

Morvan syndrome, also known as Morvan's fibrillary chorea, is a rare paraneoplastic neurological syndrome presenting with central nervous system (CNS) symptoms, peripheral nerve hyperexcitability, and autonomic nervous system (ANS) manifestations. The etiology and severity of the disease are not well understood. An adult female presented with a sudden onset of chest pain, unilateral extremity weakness, blepharospasms, and muscle spasms, with positive voltage-gated potassium channel (VGKC) antibody and positive neuronal antibody (amphiphysin) in serum. Morvan syndrome can be diagnosed in patients with myokymia, positive VGKC antibody, and neuropsychiatric symptoms with a high clinical index of suspicion. This atypical presentation of Morvan syndrome in a female identifies a novel association of amphiphysin positivity in this rare disease.

## Introduction

Morvan syndrome, also known as Morvan's fibrillary chorea, is a rare paraneoplastic neurological syndrome first identified in 1890, presenting with central nervous system (CNS) symptoms, peripheral nerve hyperexcitability, and autonomic nervous system (ANS) manifestations [1]. The etiology of Morvan syndrome is not well understood, and few reports have identified the variability in presentation. Commonly found in males, Morvan syndrome can present with diverse presentations. Patients can present with muscle fasciculation, myokymia, insomnia, hallucinations, agitations, excessive sweating, drooling, severe constipation, arrhythmias, hypertension, syndrome of inappropriate antidiuretic hormone secretion (SIADH), areflexia, and neuropathic pain [1]. Oligoclonal bands, thyrotoxicosis, thymoma, and elevated levels of gold, manganese, and mercury are associated with the disease [1]. Autoantibodies have been identified in Morvan syndrome, such as autoantibodies to voltage-gated potassium channels (VGKC), acetylcholine (ACh) receptor antibodies, contactin-associated protein 2 (CASPR2) antibodies, and leucine-rich glioma-inactivated protein 1 (LGI1) antibodies [1,2]. The prognosis of Morvan syndrome is variable. Patients can go into complete remission or are in need of continued long-term care and management. Reports have indicated that plasma exchange, intravenous immunoglobulin (IVIG), or long-term immunosuppression therapy has been used in disease management [3]. Thymoma presentation with Morvan syndrome is indicative of a poor prognosis [1].

In this study, we report a patient with an atypical presentation of Morvan syndrome. Characteristic features such as myokymia, muscle fasciculations, and positive VGKC were identified. A novel association with Morvan syndrome has been identified with positive amphiphysin antibodies in this patient with an acute onset of disease. This article was previously presented as an oral presentation and poster at the 2024 Ross Med Day at California Hospital Medical Center on May 31, 2024.

## Case presentation

The patient is a 43-year-old obese Latinx female with a history of cholecystectomy; she presented to the emergency department with her husband with sudden and acute onset of chest pain, shortness of breath, nausea, vomiting, lethargy, blurry vision in the right eye, right-sided facial and right upper extremity paresthesia, and right hemifacial spasms. The patient was in the grocery store when she started experiencing immediate symptoms. She denied any prior similar episodes. Code neuro was initiated, computed tomography (CT) and magnetic resonance imaging (MRI) of the brain were unremarkable, and cerebrovascular accident was ruled out. Laboratory studies were significant for microcytic anemia and mild thrombocytosis with iron level of 21 µg/dL, hemoglobin of 7.8 g/dL, hematocrit of 25.8%, mean corpuscular volume (MCV) of 64.6 fL, and platelet count of 437,000/µL. Physical examination was notable for right-sided weakness and decreased sensation on the right side of the face. The patient was admitted for further evaluation.

The patient was afebrile with no clinical indications of infection and continued to have chest pain reproducible with palpation. The patient developed right-sided blepharospasm, myokymia, headache, fasciculations, muscle twitching, and fatigue and was unable to ambulate. Physical examination was significant for right-sided facial swelling, the loss of sensation, and posterior neck tenderness. Right upper and lower extremity strength was 3/5, and left upper and lower extremity strength was 4/5; strength was waxing and waning throughout her hospitalization. The abdominal examination was positive for left lower quadrant tenderness. Troponins were negative. Electrocardiogram (ECG) and echocardiography were unremarkable, with an ejection fraction (EF) of 55%. Duplex ultrasound of the carotid arteries was normal. CT of the abdomen and pelvis was significant for mildly enlarged anteverted uterus, mildly heterogenous myometrium, and thick-walled hypodense 2.0 cm mass along the right superior uterine fundus of uncertain significance. Mass was not seen on follow-up transabdominal and transvaginal ultrasound given suboptimal visualization, but uterus and endometrial measurements were within normal limits (Table 1). An irregular electroencephalogram (EEG) presented features of a slow background with some anterior and posterior gradients. There was no left-right asymmetry, sharp waves, spikes, or rhythmic activity seen on EEG. Photic stimulation did not produce any abnormal activity, and EEG was negative for seizures on one tracing. The patient was administered clonazepam; however, symptoms did not improve, and clonazepam was discontinued.

**Table 1:** Imaging studies, in chronological order CT, computed tomography; CXR, chest X-ray; MRI, magnetic resonance imaging; S/P, status post

Imaging Studies	Finding
CT of the head without contrast	Negative
CT angiography of the head and neck	Negative
CXR	Negative
Extracranial arterial duplex bilateral ultrasound	Negative
MRI of the brain with + without contrast	Negative
Abdominal ultrasound	S/P cholecystectomy. Mild fatty infiltration of the liver
Maxillofacial CT with contrast	Mild stranding of subcutaneous fat overlying the chin. No fracture. Unremarkable
Follow-up MRI without contrast	Negative. No interval change
Follow-up CXR	Negative. No acute cardiopulmonary process
CT of the chest, abdomen, and pelvis with contrast	Normal thyroid gland. Mildly enlarged. Mildly heterogenous myometrium. Thick-walled hypodense 2.0 cm mass along the right superior uterine fundus. S/P cholecystectomy
Left upper extremity venous ultrasound	Negative
Pelvic ultrasound	The uterus is anteverted, measures 11.4 × 4.5 × 6.1 cm. The endometrium measures 7 mm in thickness, within normal limits. Visualized mass on prior CT not seen on ultrasound given suboptimal visualization. Negative

Of note, the patient's liver function tests trended up and improved with supportive care. Trigeminal neuralgia was ruled out due to a lack of infectious presentation and no improvement with the administration of acyclovir.

During hospitalization, the patient experienced new-onset hallucinations, which resolved spontaneously after one day. Lumbar puncture was conducted and presented with elevated WBC, segmented neutrophils, polynuclear cells, and glucose levels (Table 2). CSF culture, gram stain, and cryptococcus antigen were negative. The patient was administered Keppra, and blepharospasm and fasciculations mildly improved. A maxillofacial CT was conducted with no significant findings. Dexamethasone was administered, and the patient completed a three-day course with mild improvement. The patient continued to have chest pain, and a repeat chest X-ray (CXR) was insignificant. Continued investigations revealed positive VGKC antibodies and low positive neuronal antibodies (amphiphysin) (Table 3). IVIG was administered, and a six-day course was completed with significant improvement in strength, and the patient ambulated with assistance. In addition, the patient received one-time rituximab therapy. Throughout the hospital course, the patient was on room air, with mild hypotension, with the remainder of vitals within normal limits, and imaging studies showed no significant findings (Table 1). The patient was discharged home with family, tolerating diet, ambulatory with a front wheel walker, with planned follow-up management.

**Table 2:** CSF results

Spinal Fluids	Patient Values	Reference Ranges
CSF color	Colorless	Colorless
CSF clarity	Clear	Clear
CSF WBC	10 cells/mm^3 ^(elevated)	<5 cells/mm^3^
CSF segments	50% (elevated)	<6%
CSF lymphocyte	25% (decreased)	40%-80%
CSF monocyte	25% (normal)	15%-45%
CSF polynuclear cell	10% (elevated)	0%-2%
CSF glucose	71 mg/dL (elevated)	40-70 mg/dL
CSF protein	23 mg/dL (normal)	14-40 mg/dL

**Table 3:** Serum antibody results SOX1A, Sry-like high mobility group box 1A; NMDA, N-methyl-D-aspartate; CASPR2, contactin-associated protein 2; AMPA, α-amino-3-hydroxy-5-methyl-4-isoxazolepropionic acid; GABA-BR, gamma-aminobutyric acid B receptor; DPPX, dipeptidyl-peptidase-like protein 6; ITPR1, inositol trisphosphate receptor-1; IgLON5, immunoglobulin-like cell adhesion molecule 5; mGluR1, metabotropic glutamate receptor 1; GABA-AR, gamma-aminobutyric acid A receptor

Antibody (Serum)	Result
Neuronal antibody (amphiphysin)	Low positive A
Voltage-gated potassium channel antibody (Ab)	Positive
SOX1A antibody	Negative
Purkinje cell/neuronal nuclear IgG	Negative
Glutamic acid decarboxylase antibody	Negative
Aquaporin-4 receptor antibody	Negative
CV2.1 antibody IgG	Negative
P/Q-type calcium channel antibody	Negative
Myelin oligodendrocyte glycoprotein (MOG) antibody	Negative
Ganglionic acetylcholine receptor Ab	Negative
NMDA receptor antibody	Negative
CASPR2 antibody IgG	Negative
Leucine-rich glioma-inactivated protein 1 (LGI1) antibody	Negative
AMPA antibody	Negative
GABA-BR antibody	Negative
DPPX antibody	Negative
ITPR1 antibody	Negative
IgLON5 antibody	Negative
mGluR1 antibody	Negative
GABA-AR antibody	Negative

## Discussion

Morvan syndrome was diagnosed in a 43-year-old female with sudden onset of unilateral numbness, blepharospasms, myokymia, muscle spasms, and chest pain. Morvan syndrome commonly presents in males and is associated with myokymia, increased manganese levels, thymoma, CASPR2 antibodies, LGI1 antibodies, ACh receptor antibodies, and oligoclonal bands on CSF [1]. However, this patient's presentation of Morvan syndrome was atypical due to the sudden onset of symptoms and the lack of features of thymoma, CASPR2 antibodies, and LGI1 antibodies that are commonly associated with Morvan syndrome. The significance of this patient's atypical presentation was the novel finding of low positivity of amphiphysin and VGKC antibody, which has not been reported in the literature. Amphiphysin is commonly associated with stiff-person syndrome, paraneoplastic syndrome, myelopathy, myoclonus, encephalomyelitis, and sensory neuropathy [4].

The diagnosis of Morvan syndrome in our patient is based upon the clinical findings of myokymia, unilateral muscle spasms, hallucinations, and weakness, in addition to abnormal EEG and positive VGKC antibodies [5]. The positive amphiphysin has been described to be associated with paraneoplastic neurological syndromes [4]. Paraneoplastic neurological syndromes are generally autoimmune and can be found as a preceding finding prior to cancer diagnosis [5,6]. It is possible that this patient's positive amphiphysin suggests continued monitoring for possible cancer associations and surveillance. There are reported associations of positive amphiphysin antibodies with breast cancer and small-cell lung cancer [4,7]. EEG findings and brain imaging were nonspecific, a finding consistent with the variable presentation of Morvan syndrome [3,8].

The prognosis of Morvan syndrome is variable. Patients can go into complete remission or are in need of continued long-term care and management. It has been reported that plasma exchange, IVIG, or long-term immunosuppression therapy has been used in disease management [3]. Thymoma presentation with Morvan syndrome is indicative of a poor prognosis [1]. Our patient showed improvement in symptoms with IVIG administration but did not return to her baseline state of health prior to the acute onset.

## Conclusions

In conclusion, we report an atypical presentation of Morvan syndrome in a female with positive VGKC antibodies and novel associated positivity of amphiphysin antibodies. A high index of suspicion is required to diagnose Morvan syndrome, as the presentation of symptoms often varies, and no defining characteristic is classic for Morvan disease. This novel presentation has important implications for disease identification, management, and continued understanding of Morvan syndrome etiology. The unpredictable course of Morvan syndrome and its variable presentation have important implications for patient care and the course of outcome in this rare disease.
